# Predictors for inappropriate proton pump inhibitor use: observational study in primary care

**DOI:** 10.3399/BJGP.2022.0178

**Published:** 2022-09-21

**Authors:** Lieke Maria Koggel, Marten Alexander Lantinga, Frederike Leonie Büchner, Joost Paulus Hubertus Drenth, Jacqueline Sarah Frankema, Edwin Johannes Heeregrave, Mette Heringa, Mattijs Everard Numans, Peter Derk Siersema

**Affiliations:** Department of Gastroenterology and Hepatology, Radboud University Medical Centre, Nijmegen.; Department of Gastroenterology and Hepatology, Radboud University Medical Centre, Nijmegen; gastroenterologist, Department of Gastroenterology and Hepatology, University Medical Centres Amsterdam, Amsterdam.; Department of Public Health and Primary Care, Leiden University Medical Center, Leiden.; Department of Gastroenterology and Hepatology, Radboud University Medical Centre, Nijmegen.; Appropriate Care Project, The Dutch National Health Care Institute, Diemen.; Appropriate Care Project, The Dutch National Health Care Institute, Diemen.; SIR Institute for Pharmacy Practice and Policy, Leiden.; Department of Public Health and Primary Care, Leiden University Medical Center, Leiden.; Department of Gastroenterology and Hepatology, Radboud University Medical Centre, Nijmegen.

**Keywords:** anti-ulcer agents, dyspepsia, inappropriate prescribing, non-steroidal anti-inflammatory drugs, primary health care, proton pump inhibitors

## Abstract

**Background:**

Proton pump inhibitor (PPI) indications are limited to gastrointestinal disorders and ulcer prophylaxis. However, PPIs are among the most frequently prescribed drugs.

**Aim:**

To evaluate the appropriateness of PPI prescriptions and identify predictive factors for inappropriate PPI use.

**Design and setting:**

Observational study using a Dutch primary care database with all new PPI prescriptions between 2016 and 2018.

**Method:**

Individual patient data and details on PPI use were collected. The appropriateness of initiation and continuation of PPI prescriptions was evaluated using the applicable guidelines.

**Results:**

In total, 148 926 patients (aged ≥18 years) from 27 general practices were evaluated. A total of 23 601 (16%) patients started PPI therapy (mean age 57 [SD 17] years, 59% female). Valid PPI indications at initiation were seen in 10 466 PPI users (44%). Predictors for inappropriately initiated PPI use were older age (odds ratio [OR] 1.03, 95% confidence interval [CI] = 1.03 to 1.03), and use of non-selective non-steroidal anti-inflammatory drugs (OR 5.15, 95% CI = 4.70 to 5.65), adenosine diphosphate receptor inhibitors (OR 5.07, 95% CI = 3.46 to 7.41), COX-2 inhibitors (also known as coxibs) (OR 3.93, 95% CI = 2.92 to 5.28), and low-dose aspirin (OR 3.83, 95% CI = 3.07 to 4.77). Despite an initial valid indication, PPI use was inaccurately continued in 32% of patients on short-course therapy for dyspepsia and in 11% of patients on ulcer prophylaxis.

**Conclusion:**

More than half of PPI users in primary care were found to have an inappropriate indication, with unnecessary ulcer prophylaxis related to drug use being one of the leading causes. Future initiatives to reduce PPI use for unnecessary ulcer prophylaxis and timely deprescription if PPI is no longer indicated, are needed.

## INTRODUCTION

Proton pump inhibitors (PPIs) are among the most prescribed drugs worldwide and are the cornerstone for treating and preventing acid-related disorders.^1^^–^^4^ There use has a major impact as it accounts for at least 37 million euros spent on health care annually in the Netherlands alone.^5^

PPI therapy is frequently prescribed in the absence of an appropriate indication.^6^ Examples of inappropriate PPI use are ulcer prophylaxis in patients without risk factors (for example, steroid therapy alone) and overtreatment of functional dyspepsia.^6^ Inappropriate PPI use may potentially harm patients through adverse drug reactions or drug–drug interactions. Increasing evidence shows that long-term PPI use is associated with severe adverse drug reactions, such as *Clostridium difficile* colitis *,* malabsorption of magnesium, osteoporosis, and kidney disease.^7^^–^^10^

Prior studies have identified unnecessary PPI use for ulcer prophylaxis (that is, in patients without risk factors) as an important factor associated with inappropriate PPI therapy.^11^^,^^12^ However, use of certain drugs or clinical conditions that are most predictive for inappropriate PPI use in clinical practice remain largely unknown, which hampers targeted interventions to reduce PPI use. This study aimed to evaluate the appropriateness of PPI therapy in a large primary care setting in the Netherlands and determine predictors for inappropriate PPI use.

## METHOD

This study uses real-world, pseudonymised, routine primary care data covering the Leiden/The Hague region in the Netherlands. Continuous updated electronic medical record data from all patients (aged ≥18 years) from GP centres in the Extramural LUMC Academic Network (ELAN) were accessible. A total of 27 general practice centres associated with ELAN (each with 2–6 practising GPs) could be approached for this study, covering 148 926 patients. All practice centres associated with ELAN use an ‘informed opt-out’ procedure, so electronic medical record data of the patients enlisted with these practices can be used for research purposes. No more than 5% of all patients chose informed opt-out. The general practice centres can be characterised as representative for the average Dutch population, randomly spread over rural, suburban, and highly urbanised areas. According to the Dutch healthcare system, all residents primarily contact their GP in the case of a health problem. GPs can deal with routine health issues, including upper gastrointestinal disorders. If indicated, GPs can refer their patient to a specialist.

**Table:** How this fits in

Although overuse of proton pump inhibitors (PPIs) is a common issue worldwide, predictors for this remain insufficiently known. This observational study using real-world primary care data identified older age and non-selective non-steroidal anti-inflammatory drug use as most predictive for inappropriate PPI use. The study also showed that unnecessarily continued PPI therapy was common in patients using PPI therapy for dyspepsia or as ulcer prophylaxis. Future initiatives on reducing inappropriate PPI use should target these patient groups.

### Data collection

The database used International Classification of Primary Care (ICPC) codes for medical conditions and Anatomic Therapeutic Chemical (ATC) codes for drug use. Available data included patient characteristics, medical history, drug prescriptions, and GP consultations. Drug prescriptions were linked to the pharmacist’s database in which all pharmacy data from participating GP practices is stored. Therefore, drug prescriptions included all drugs prescribed by GPs and non-prescription medicine in case this was registered by pharmacies. All patients with PPI prescriptions and upper gastrointestinal symptoms or conditions were identified using ATC and ICPC codes (see Supplementary Boxes S1 and S2). The accuracy of ICPC code registration in Dutch general practices is around 90%.^13^ Data between 2015 and 2018 were available. New PPI usage periods were identified over the years 2016–2018. Data in 2015 were used to confirm that PPI prescriptions were initiated between 2016 and 2018, defined as no PPI use during at least 3 months before the start of the new PPI prescription.

### Drug prescription variables

Drug usage periods were calculated by merging repeat (refill) prescriptions. The usage periods of drugs that are known for chronic use or as a treatment during a predefined period of time (corticosteroids, anticoagulants, antidepressants, and spironolactone) were defined as the start date of the first prescription until the end date of the last prescription. For drugs that are potentially used short term (PPIs, H_2_-blockers, antacids, *Heliobacter pylori* [ *H. pylori* ] eradication therapy, and non-steroidal anti-inflammatory drugs [NSAIDs]), a unique usage period was created if the interval between two prescriptions exceeded 3 months. For example, if the interval between the end date of prescription 1 and the start date of prescription 2 was >3 months, the end date of prescription 1 was considered the end date of the first usage period and the start date of prescription 2 as the start date of the second usage period.

End dates of a drug prescription were calculated using the start date, dosage, and usage frequency. To categorise drug prescriptions that did not specify an exact frequency, the lowest possible usage frequency was selected (for example, ‘one to three times daily’ was transformed into ‘one time daily’). Furthermore, on-demand use was converted to one-third of the time used (for example, ‘one time daily, on demand’ was transformed to ‘one time daily, every 3 days’). If the prescribed frequency was not provided, it was replaced by once daily. Finally, if no dosage was available, prescriptions were considered to end after 3 months.

Chronic PPI use was defined as >180 defined daily doses (DDDs)/year, a technical unit measuring drug consumption, as a proxy of >6 months PPI use.^14^^,^^15^ NSAID prescriptions were recorded as high dose if the DDD was exceeded. Lastly, *H. pylori* eradication therapy was defined as either a fixed-dose combination or the prescription of a PPI with at least two types of antibiotics initiated simultaneously.

### Appropriateness of PPI therapy

Appropriateness of PPI therapy was assessed for all patients receiving a new PPI prescription between 2016 and 2018. In case of multiple PPI usage periods in a single patient, appropriateness of PPI use was categorised based on the earliest PPI usage period and scored according to the Dutch College of General Practitioners guideline ‘Upper gastrointestinal symptoms’ (version 2013) and clinical decision rules.^16^^–^^18^ PPI therapy was deemed appropriate if used for

- confirmed gastroesophageal reflux disease;
- peptic ulcer disease (if registered <3 months before start of PPI);
- short-course therapy for dyspepsia (if registered <6 months before start of PPI);
- alarm symptoms (for example, haematemesis) if registered <1 month before start of PPI; and
- as part of eradication therapy for *H. pylori.*^18^

Furthermore, PPI use was determined appropriate for ulcer prophylaxis in high-risk patients when using NSAIDs, low-dose aspirin, or in patients with a history of peptic ulcer disease. To assess if a patient was at high risk of developing gastroduodenal ulcers their age, comorbidities, and concomitant drug use at the time of PPI prescription was evaluated. Chronic use of PPIs is only indicated for severe reflux oesophagitis, Barrett’s oesophagus, Zollinger–Ellison syndrome, and chronic ulcer prophylaxis.^19^^,^^20^ Indications were evaluated based on registered ICPC and ATC codes. ICPC codes of medical conditions such as reflux oesophagitis are only used if confirmed by additional examination such as a gastroscopy. In cases where a medical condition is not confirmed, symptom ICPC codes such as pyrosis were used. Supplementary Box S2 shows all valid PPI indications, including corresponding ICPC and ATC codes.

### Appropriate duration of PPI therapy

The accepted duration of PPI use for a temporary indication to treat upper gastrointestinal disorders was limited to 3 months. These include short-course PPI therapy for dyspepsia, treatment of peptic ulcer disease, alarm features such as haematemesis, and *H. pylori* eradication. If a PPI was started as ulcer prophylaxis, it had to be stopped within 3 months after cessation of the drug that initiated PPI use.

### Predictors for inappropriate PPI use

To identify predictive factors for inappropriate PPI use, users of PPIs (PPI use group) were compared with non-users of PPIs (non-PPI use group) who had consulted the GP for upper gastrointestinal conditions as a control group. Factors in the regression model included patient characteristics (for example, age, sex, and body mass index [BMI]), comorbidities (for example, diabetes mellitus and heart failure), anti-reflux medication used before the start of PPIs, and concomitant drug use associated with PPI indications. To allow comparison between inappropriate use in the PPI group and the non-PPI use group, concomitant drug use in the inappropriate PPI use group was not restricted to a specific time interval between 2016 and 2018. This means that all concomitant drug use in the period of 2016–2018 was included regardless of the duration of use or, for the inappropriate PPI use group, interval between PPI use and concomitant drug use.

### Statistical analysis

Normally distributed data were presented as mean (standard deviation [SD]) and non-normally distributed data as median and interquartile range. χ^2^-testing was performed to compare categorical variables. Patients were clustered within practices and, therefore, a mixed-model logistic regression was used to determine predictive factors for inappropriate PPI use. A random intercept model was performed with the other factors fixed. Variables with a *P*-value <0.2 in the univariate analysis were included in the multivariate analysis. A backwards model was used to stepwise eliminate the variables with the highest *P*-value until all variables in the model had a *P*-value <0.05. Two-sided testing with a *P*-value of <0.05 was considered significant. IBM SPSS Statistics (version 25.0) and R (version 4.1.1) packages *haven*, *funnelR*, and *ggplot2* were used to process and analyse the data.

## RESULTS

### PPI prescriptions and patient characteristics

**Figure 1.**
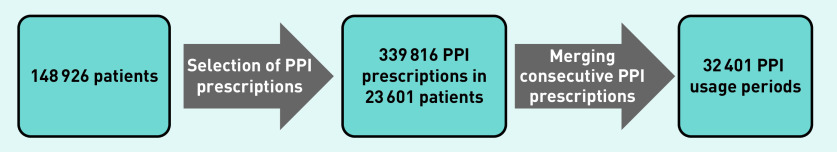
*Selection of PPI usage periods in 2016–2018. PPI = proton pump inhibitor.*

In total, 339 816 new PPI prescriptions between 2016 and 2018 in 23 601/148 926 patients (16%) were identified. Merging consecutive (refill) prescriptions resulted in 32 401 PPI usage periods (Figure 1). The prescribed frequency and dosage were not provided in 3190 (1%) and 36 (0.01%) PPI prescriptions, respectively. The number of new PPI usage periods was relatively stable throughout 2016–2018 (11 235 in 2016, 10 955 in 2017, and 10 211 in 2018).

**Figure 2.**
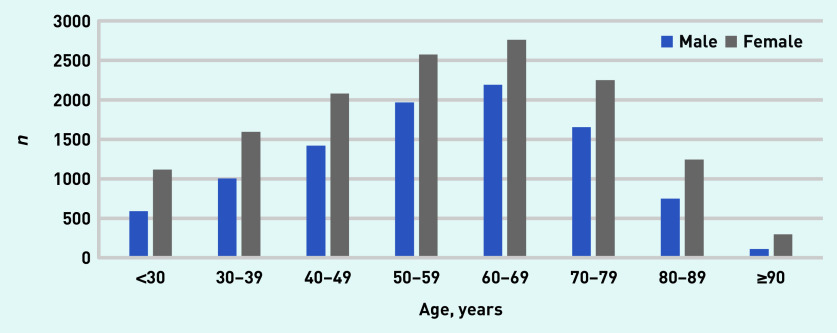
*Age and sex of patients with a PPI usage period in 2016–2018. PPI = proton pump inhibitor.*

Figure 2 shows the age and sex distribution of all patients with PPI usage periods in 2016–2018. Mean age at initiation of PPI prescription was 57 years (SD 17), of whom 59% were female (Table 1). A total of 2823 (12%) patients were registered as active smokers and 3106 (13%) as active alcohol users. Mean BMI was 28 kg/m^2^ (SD 6), and diabetes mellitus was registered in 2536 (11%) patients and heart failure in 446 (2%) patients.

**Table 1.** Patient characteristics, based on first PPI usage period per patient

**Characteristic**	**All PPI users (*n*= 23 601), *n* (%)^a^**	**Appropriate PPI users (*n*= 10 466), *n* (%)^a^**	**Inappropriate PPI users (*n*= 13 135), *n* (%)^a^**
**Sex, female**	13 916 (59)	6237 (60)	7679 (58)
**Age, years, mean (SD)**	57 (17)	61 (18)	54 (16)
**BMI, kg/m^2^, mean (SD)^b^**	28 (6)	28 (5)	28 (6)
**Current smoker**	2823 (12)	1222 (12)	1601 (12)
**Use of alcohol**	3106 (13)	1678 (16)	1428 (11)
**Diabetes mellitus**	2536 (11)	1346 (13)	1190 (9)
**Heart failure**	446 (2)	206 (2)	240 (2)
**Rheumatoid arthritis**	125 (1)	59 (1)	66 (1)
**Peptic ulcer disease**	596 (3)	483 (5)	113 (1)
**Reflux oesophagitis**	523 (2)	523 (5)	0 (0)
**Antacid**	884 (4)	440 (4)	444 (3)
**H_2_RA**	801 (3)	527 (5)	274 (2)
**Non-selective NSAID**	9281 (39)	4790 (46)	4491 (34)
**COX-2 inhibitor**	560 (2)	165 (2)	395 (3)
**Low-dose aspirin**	3048 (13)	1699 (16)	1349 (10)
**Vitamin K antagonist**	926 (4)	403 (4)	523 (4)
**ADP receptor inhibitor**	1030 (4)	393 (4)	637 (5)
**DOAC**	513 (2)	201 (2)	312 (2)
**LMWH**	845 (4)	436 (4)	409 (3)
**Systemic corticosteroid**	1943 (8)	825 (8)	1118 (9)
**SSRI**	1565 (7)	804 (8)	761 (6)
**Trazadone**	43 (0.2)	23 (0.2)	20 (0.2)
**Venlafaxine**	279 (1)	151 (1)	128 (1)
**Duloxetine**	54 (0.2)	20 (0.2)	34 (0.3)
**Spironolactone**	285 (1)	106 (1)	179 (1)

a *Unless otherwise stated.*

b n *=14 764 missing. ADP = adenosine diphosphate. BMI = body mass index. DOAC = direct-acting oral anticoagulant. H_2_RA = H_2_ receptor antagonist. LMWH = low-molecular-weight heparin. NSAID = non- steroidal anti-inflammatory drug. PPI = proton pump inhibitor. SSRI = selective serotonin reuptake inhibitor.*

At the time of PPI prescription, 9281 (39%) patients used non-selective NSAIDs and 3048 (13%) patients used low-dose aspirin. Antacids were prescribed before the start of PPI in 884 (4%) and H_2_-blockers in 801 (3%) patients.

In 16 328 (69%) patients, PPIs were prescribed for <3 months. A total of 6794 (29%) patients in the PPI use group fulfilled the criteria for chronic PPI use.

### Appropriateness at start of PPI therapy

**Figure 3.**
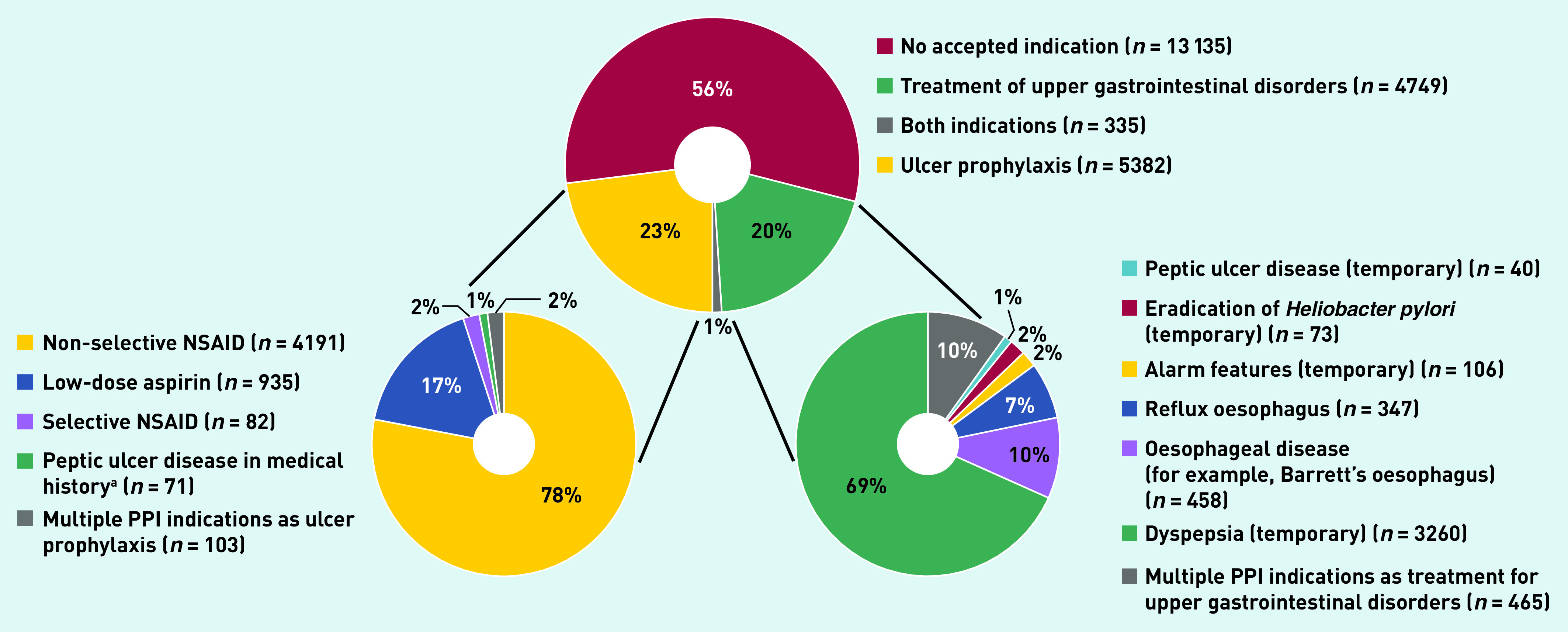
*PPI indications. ^a^If not already in combination with NSAID or low-dose aspirin usage. NSAID = non-steroidal anti-inflammatory drug. PPI = proton pump inhibitor.*

A total of 10 466 (44%) patients had an appropriate indication for PPI use at the start of PPI therapy. The indications for PPI use was equally distributed between treatment for upper gastrointestinal conditions (*n* = 4749, 20%) and ulcer prophylaxis (*n* = 5382, 23%). Table 2 and Figure 3 show the indications for appropriately prescribed PPIs. Dyspepsia was the leading upper gastrointestinal symptom in patients with a PPI (*n* = 3260, accounting for 69% of PPIs started as treatment of upper gastrointestinal disorders). Use of non-selective NSAIDs and low-dose aspirin use were responsible for 78% (*n* = 4191) and 17% (*n* = 935) of ulcer prophylaxis indications, respectively.

**Table 2.** PPI indications

**Variable**	**PPI users (*n*= 23 601), *n* (%)**
**Treatment of upper gastrointestinal disorders**	**4749 (20)**
Temporary indication	
Dyspepsia	3260 (14)
Peptic ulcer disease	40 (0.2)
Alarm features (for example, haematemesis)	106 (0.4)
Eradication of *Heliobacter pylori*	73 (0.3)
Chronic indication	
Oesophageal disease (for example, Barrett’s oesophagus)	458 (2)
Reflux oesophagitis	347 (1)
Multiple PPI indications as treatment of upper gastrointestinal disorders	465 (2)

**Ulcer prophylaxis**	**5382 (23)**
Non-selective NSAID	4191 (18)
Low-dose aspirin	935 (4)
COX-2 inhibitor	82 (0.3)
Peptic ulcer disease in medical history^a^	71 (0.3)
and usage of coumarins	10 (0.04)
and usage of DOAC	6 (0.03)
and usage of LMWH	2 (0.008)
and usage of ADP receptor inhibitor	13 (0.06)
and usage of thrombolytics	0 (0)
and usage of systemic corticosteroid	15 (0.06)
and usage of SSRI	8 (0.03)
and usage of venlafaxine	0 (0)
and usage of duloxetine	0 (0)
and usage of trazodone	0 (0)
and usage of spironolactone	3 (0.01)
overlay in comedication use	14 (0.06)
Multiple PPI indications as ulcer prophylaxis	103 (0.4)

**Both treatment of upper gastrointestinal disorders and ulcer prophylaxis**	**335 (1)**

**No accepted indication**	**13 135 (56)**

a *If not already in combination with NSAID or low-dose aspirin usage. ADP = adenosine diphosphate. DOAC = direct- acting oral anticoagulant. LMWH = low-molecular-weight heparin. NSAID = non-steroidal anti- inflammatory drug. PPI = proton pump inhibitor. SSRI = selective serotonin reuptake inhibitor.*

An inappropriate PPI indication was identified in 13 135 (56%) patients. In this group, 8493 patients (65%) used drugs associated with an indication as ulcer prophylaxis at the time of PPI prescription. These drugs primarily included non-selective NSAIDs (34%), low-dose aspirin (10%), and systemic corticosteroids (9%) (Table 1).

Appropriateness of PPI therapy ranged from 47% to 67% between general practices (*P*<0.001, Supplementary Table S1 and Supplementary Figure S1).

### Appropriate duration of PPI therapy

Of patients receiving a short course of PPI for dyspepsia, 1042/3260 (32%) did not stop PPI treatment within 3 months. In 3944/5717 (69%) PPI users with an appropriate indication for PPI as ulcer prophylaxis, the drug that initiated PPI treatment was stopped during follow-up. Despite stopping, 446 (11%) patients continued PPI use for >3 months. This included 311 (70%) patients that used a PPI as ulcer prophylaxis for non-selective NSAIDs use, 118 (26%) for low-dose aspirin use, 5 (1%) for COX-2 inhibitor (also known as coxib) use, and 12 (3%) patients with a history of peptic ulcer disease combined with comedication use that is associated with a higher bleeding risk (data not shown).

### Predictors for inappropriate PPI therapy

A total of 13 135 patients in the inappropriate PPI use group were compared with 3155 patients in the non-PPI use group (see Supplementary Table S2). Variables with substantial missing data (BMI), not fully registered data (smoking, alcohol use, and antacid and H_2_-blocker use), small numbers (rheumatoid arthritis, peptic ulcer disease, and spironolactone), or a direct association with an appropriate PPI indication (reflux oesophagitis) were excluded.

Predictors for inappropriate PPI use were age (odds ratio [OR] 1.03 increment per year, 95% confidence interval [CI] = 1.03 to 1.03) and drug use associated with PPI indications (Table 3). Non-selective NSAID use (OR 5.15, 95% CI = 4.70 to 5.65) and adenosine diphosphate receptor inhibitor use (OR 5.07, 95% CI = 3.46 to 7.41) had the strongest association with inappropriate PPI use, followed by COX-2 inhibitor use (OR 3.93, 95% CI = 2.92 to 5.28) and low-dose aspirin use (OR 3.83, 95% CI = 3.07 to 4.77).

**Table 3.** Mixed-model multivariate logistic regression analysis for inappropriate PPI use

**Category**	**Univariate logistic regression**		**Multivariate logistic regression**	
	**OR**	**95% CI**	**OR**	**95% CI**
**Sex, male**	1.13	1.04 to 1.23	—	—
**Age, increment per year**	1.03	1.03 to 1.03	1.03	1.03 to 1.03
**Diabetes mellitus**	1.58	1.35 to 1.84	—	—
**Heart failure**	1.37	1.08 to 1.75	—	—
**Non-selective NSAID**	3.44	3.16 to 3.74	5.15	4.70 to 5.65
**COX-2 inhibitor**	4.92	3.70 to 6.54	3.93	2.92 to 5.28
**Low-dose aspirin**	5.31	4.32 to 6.52	3.83	3.07 to 4.77
**Vitamin K antagonist**	1.97	1.57 to 2.49	—	—
**ADP receptor inhibitor**	8.57	5.94 to 12.35	5.07	3.46 to 7.41
**DOAC**	3.55	2.56 to 4.92	2.54	1.80 to 3.57
**LMWH**	4.58	3.50 to 5.99	2.91	2.20 to 3.85
**Systemic corticosteroids**	3.08	2.69 to 3.53	2.37	2.05 to 2.74
**SSRI**	1.66	1.41 to 1.96	1.77	1.49 to 2.11
**SNRI**	2.37	1.66 to 3.38	2.18	1.49 to 3.19

*ADP = adenosine diphosphate. DOAC = direct-acting oral anticoagulant. LMWH = low-molecular-weight heparin. NSAID = non-steroidal anti-inflammatory drug. OR = odds ratio. PPI = proton pump inhibitor. SNRI = serotonin and norepinephrine reuptake inhibitor. SSRI = selective serotonin reuptake inhibitor.*

## DISCUSSION

### Summary

This study found that, at the time of analysis, more than half of PPIs prescribed in primary care were not adequately indicated. The most important predictors for inappropriate PPI therapy were age and the use of drugs, such as non-selective NSAIDs, for which ulcer prophylaxis is only indicated in high-risk patients. One-third of PPIs started as short-course therapy for dyspepsia and one-tenth of PPIs started as ulcer prophylaxis were continued after the indication was no longer valid.

### Strengths and limitations

The data used were obtained directly from electronic medical records without a pre-known research purpose. Using real-world data allows accurate investigation of current clinical practice. Moreover, a strength of this study is the large size, which allowed a detailed assessment of PPI appropriateness. Also, as most PPIs are prescribed in a primary care setting in the Netherlands, this population is representative for assessing the appropriateness of PPI use.^18^

This study is, however, limited by its retrospective design. First, patient characteristics, registered comorbidity, and comedication prescriptions were used as a proxy to determine appropriateness of PPI use. Some assumptions were needed for comorbidity stages and duration of comedication use; however, appropriateness of PPI therapy was always given the benefit of the doubt. Second, not all anti-reflux medication use was known as non-prescription drug registration was incomplete; however, apart from including drugs prescribed by GPs, non-prescription drug use registered by pharmacists was also included. Moreover, as a proxy for general non-PPI users, non-PPI users who consulted the GP for upper gastrointestinal conditions were used as a control group for the logistic regression analysis. Nonetheless, two groups without valid PPI indication were compared to determine possible predictors for inappropriate PPI use. Furthermore, some variables, such as BMI and alcohol use, could not be included in the logistic regression analysis because of missing data. Finally, total duration of PPI use and number of patients with chronic use may have been underestimated as there was only access to data up to 2018.

### Comparison with existing literature

The high percentage of inappropriately initiated PPI prescriptions (56%) corresponds with an earlier Dutch study.^21^ In contrast to the current study, those authors had no access to electronic primary care patient records, which may potentially overestimate the number of inappropriate users of PPI. Another study from Denmark had similar access to primary care source data and showed that 25% of patients had an invalid PPI indication.^22^ However, appropriateness could have been overestimated in that study as all patients using NSAIDs or aspirin were considered as appropriate users of PPI. Moreover, bias could have been introduced as the prescribing physicians collected the data themselves, which was not the case in the current study as real-world data was extracted by authors with no role in prescribing this PPI therapy.

Patients using non-selective NSAIDs were identified as having the highest odds for inappropriate PPI use. This finding is in line with previous studies.^11^^,^^12^^,^^21^ A questionnaire study showed that inappropriate PPI therapy as ulcer prophylaxis was recommended by 35% of GPs and internists when starting NSAIDs in low-risk patients.^23^ As non-selective NSAIDs are frequently prescribed, its use in low-risk patients is likely one of the leading causes of inappropriate PPI use.^24^

Moreover, patient age was found to be predictive for inappropriate PPI therapy. This could be related to the increasing number of drugs patients use when age increases.^25^ A previous study showed that the number of drugs used was a predictor of inappropriate PPI therapy in older people.^26^ A possible explanation could be that physicians tend to prescribe ulcer prophylaxis more often in frail older people regardless of a valid indication.^27^

Another important risk factor for inappropriate PPI use is unjustified continuation of temporary indicated PPIs in patients with dyspepsia or as ulcer prophylaxis, as already suggested by prior studies.^28^^,^^29^ Not explicitly informing patients that PPI treatment is of limited duration and lack of physician follow-up may lead to unjustified continuation of PPI therapy. Furthermore, rebound symptoms may complicate discontinuation of PPI use and, in the case of dyspepsia, suggesting an alternative therapy such as lifestyle measures to patients can be challenging.^30^^,^^31^

### Implications for practice

By identifying predictors for PPI overuse, the current study provides possible targets for future interventions to reduce inappropriate PPI use. Previous studies have shown different interventions that successfully reduce inappropriate PPI use, such as prescriber and patient education, PPI use evaluation, and self-management plans.^21^^,^^32^^,^^33^ However, sustainable and time-efficient strategies are lacking. One potential strategy is a close collaboration between GPs and pharmacists to double-check PPI indications and to stress discontinuing inappropriate PPI therapy. Furthermore, GPs could also play a role when they notice that PPIs are inappropriately prescribed by medical specialists in secondary care.

## Data

Data are available from the corresponding author on reasonable request.
